# Intensivist-led ultrasound-guided percutaneous tracheostomy: a phase IV cohort study

**DOI:** 10.5935/2965-2774.20230174-en

**Published:** 2023

**Authors:** Pedro Fortes Osório Bustamante, Bruno Adler Maccagnan Pinheiro Besen, Amanda Pinto Botêga, Filipe Matheus Cadamuro, Marcelo Park, Pedro Vitale Mendes, Roberta Muriel Longo Roepke

**Affiliations:** 1 Intensive Care Unit, Hospital das Clínicas, Faculdade de Medicina, Universidade de São Paulo - São Paulo (SP), Brazil

**Keywords:** Analgesia/sedation, Mechanical ventilation, Neurocritical care, Perioperative care, Trauma

## Abstract

**Objective:**

To describe, with a larger number of patients in a real-world scenario following routine implementation, intensivist-led ultrasound-guided percutaneous dilational tracheostomy and the possible risks and complications of the procedure not identified in clinical trials.

**Methods:**

This was a phase IV cohort study of patients admitted to three intensive care units of a quaternary academic hospital who underwent intensivist-led ultrasound-guided percutaneous tracheostomy in Brazil from September 2017 to December 2021.

**Results:**

There were 4,810 intensive care unit admissions during the study period; 2,084 patients received mechanical ventilation, and 287 underwent tracheostomy, 227 of which were performed at bedside by the intensive care team. The main reason for intensive care unit admission was trauma, and for perform a tracheostomy it was a neurological impairment or an inability to protect the airways. The median time from intubation to tracheostomy was 14 days. Intensive care residents performed 76% of the procedures. At least one complication occurred in 29.5% of the procedures, the most common being hemodynamic instability and extubation during the procedure, with only 3 serious complications. The intensive care unit mortality was 29.1%, and the hospital mortality was 43.6%.

**Conclusion:**

Intensivist-led ultrasound-guided percutaneous tracheostomy is feasible out of a clinical trial context with outcomes and complications comparable to those in the literature. Intensivists can acquire this competence during their training but should be aware of potential complications to enhance procedural safety.

## INTRODUCTION

Tracheostomy is performed in approximately 10 - 24% of patients in intensive care units (ICUs), mainly to treat patients undergoing a long period of mechanical ventilation (MV) or to secure the airway of a neurologically impaired patient.^(1,2)^

This procedure poses many advantages in the care of critically ill patients, with a relatively low risk of complications.^(3)^ With tracheostomy, there is a reduced need for analgesic and sedative medications and an increase in patient comfort; it can facilitate weaning of MV and enable faster recovery of patient autonomy;^(4,5)^ and it may provide a shorter ICU length of stay.^(6)^ While a reduction in ventilator-associated pneumonia (VAP) is cited as one of the main advantages of the procedure, this benefit is not consistent in larger and better designed studies.^(6,7)^

With the development of a percutaneous dilatation technique by Ciaglia et al. in 1985,^(8)^ the use of tracheostomy increased. The use of tools to guide the procedure began in 1995 with the introduction of the bronchoscopy-guided technique,^(9)^ and this was expanded in 1999 with the use of ultrasound.^(10)^ Our group has performed bronchoscopically guided percutaneous tracheostomies since the beginning of the year 2000;^(11)^ however, given the unavailability of bronchoscopy to guide all percutaneous tracheostomies, we started adopting the ultrasound-guided technique.^(12)^ Following the publication of the TRACHUS Trial,^(13,14)^ we have been using ultrasound-guided percutaneous tracheostomy as a standard in most intensivist-led tracheostomies. Although the percutaneous procedure can be performed both by surgeons and intensivists, there is scarce literature regarding the risk of complications and potential outcomes among patients undergoing intensivist-led tracheostomy.

Our objective is to describe, with a larger number of patients in a real-world scenario following routine implementation, intensivist-led ultrasound-guided percutaneous dilational tracheostomy and the possible risks and complications of the procedure not identified in clinical trials.

## METHODS

### Study design, setting and ethics

This was a phase IV descriptive cohort study in three ICUs in a quaternary academic hospital in São Paulo, Brazil. The total number of beds in these three ICUs varied during the period of data collection because of unit relocation and the severe acute respiratory syndrome coronavirus 2 (SARS-CoV-2) pandemic, with an average number of 38 beds. One of the ICUs admitted mainly major trauma and neurocritical patients, while the two other units were mixed ICUs taking care mainly of medical and neurocritical patients. All of them received emergency surgical patients according to the hospital needs. Following the publication of the TRACHUS trial,^(13)^ we maintained a prospective quality assessment database, with data from September 2017 to December 2021. Because of the study design - retrospective analysis of a prospective quality improvement database, the application of informed consent was waived by the institutional review board (CAAE: 61006622.8.0000.0068).

### Study population and outcomes

We included all patients submitted to a bedside percutaneous tracheostomy performed by intensive care physicians. Patients submitted to a surgical tracheostomy or whose tracheostomy was performed by a surgical team were excluded.

The main outcome of interest was the occurrence of adverse events related to the procedure. We also collected clinical outcomes after tracheostomy, such as weaning, ICU and hospital mortality and length of stay. Adverse events prespecified were those commonly reported in the literature and were categorized as “Adverse events during the procedure”, which included hemodynamic instability, extubation during the procedure, cuff puncture, desaturation, incomplete procedure, surgical technique conversion and bleeding; “Infections”, which included stoma infection, VAP in 48 hours after the procedure and mediastinitis; “Airway lesions”, which included posterior tracheal wall puncture, pneumothorax or pneumomediastinum, false passage, tracheoesophageal fistula, tracheal stenosis and tracheo-innominate artery fistula; and “Other complications”, which included atelectasis and premature decannulation.

### Procedural indications and characteristics

The consultant intensivist in charge of the patient prescribed the tracheostomy. The intensivists performed the procedure after obtaining informed consent from the patient or their surrogate. Our ICUs usually do not undertake routine early tracheostomy. The decision for bedside tracheostomy performed by the intensivist or the selection of surgical tracheostomy is also made by the intensivist after close examination of the patient. Overall, the main indication for referral to a surgical team is an expected anatomically difficult procedure, which mainly occurs when the patient cannot be positioned with hyperextension of the neck. At the beginning of the coronavirus disease 2019 (COVID-19) pandemic, because of work overload in ICUs, some procedures were referred to surgical teams and thus were excluded from this study.^(15)^

Before the procedure, bedside neck ultrasound performed by the intensive care team to search for possible contraindications for the procedure (i.e., a large thyroid or large vessels in the line of the puncture) is encouraged.

Tracheostomy is performed at the bedside, usually by a critical care fellow or 1st year general surgery resident. In all such procedures, a consultant intensivist trained in percutaneous tracheostomy directly supervises the physicians-in-training.

In this study, all procedures were performed with real-time ultrasound guidance. The preferable site of puncture was between the second and third tracheal rings, with the patient positioned in the dorsal decubitus position with hyperextension of the neck. Local anesthesia with a vasoconstrictor was always administered at the intended puncture site and tract. After tracheal puncture, a guide wire was placed inside the trachea according to Seldinger’s technique. A cutaneous incision was then performed, followed by tract dilation of the subcutaneous tissue and tracheal puncture site. Final tracheal dilation was performed either with Griggs forceps or a single dilatator, according to the kit availability in the hospital at the time of the procedure. After tracheal dilatation, the cannula was placed, the guide-wire was removed, and correct placement of the cannula was checked through thorax movement, ventilator curves and maintenance of adequate oxygenation. The cannula was fastened with a proper instrument, and the procedure was finished. After the procedure, both lung ultrasound and a chest X-ray were performed to check the cannula placement and to search for possible complications (pneumothorax and pneumomediastinum).

All procedures were performed with continuous physiological monitoring and under intravenous general anesthesia and neuromuscular blockade at the discretion of the intensivist in charge of the procedure. Further details of the technique are described in a previously published manuscript.^(12)^

### Data collection and variables

The characteristics of the patients, diagnosis at admission, indication and timing of the procedure, number of extubation attempts before the decision to perform the tracheostomy, and characteristics and complications of the procedure were collected prospectively. The outcomes of the patients, both in the ICU and in the hospital, were also recorded. In cases of missing data, the authors retrospectively searched the medical records of the patients. When information was missing from the medical records, no imputation was made. All data were collected in REDCap Software.^(16,17)^

### Definitions

The procedural complications were defined a priori. Hemodynamic instability was defined as the need to start or increase the dose of vasopressors. Extubation during the procedure was defined as the loss of tracheal intubation before the insertion of the tracheostomy tube. Desaturation was defined as any episode of hypoxemia (oxygen saturation below 90%) during the procedure. Bleeding was defined as any bleeding that needed intervention (surgical or transfusion). If the procedure could not be completed, it was defined as incomplete. If the intensivist team decided to change the percutaneous technique to a surgical technique during the procedure, it was defined as a surgical technique conversion. Pneumothorax and pneumomediastinum diagnosed immediately after the procedure were considered procedure-related complications. Ventilator-associated pneumonia was recorded as a procedure-related complication if the symptoms started within 48 hours after the tracheostomy was performed and antimicrobial therapy was prescribed; the definition of VAP followed local guidelines, which include a combination of new-onset SIRS criteria, new pulmonary infiltrates, worsening tracheal secretions and worsening gas exchange, with or without microbiological confirmation. The use of antibiotics in the first 48 hours after the procedure was checked to minimize underreporting of this complication. Any incidental decannulation in the first 7 days after the procedure was considered premature decannulation. There was no period limitation for the definition of stoma infection and mediastinitis, as there was for tracheosophageal fistula, tracheal stenosis and trachea-innominate artery fistula, although follow-up for these complications was performed only during the index hospitalization. Bronchoscopy, upper endoscopy or neck tomography were performed only if symptoms of airway lesions justified ordering these exams.

### Statistical analysis

Data were processed and analyzed using R free source software^(18)^ using RStudio IDLE (version 1.4.1717).^(19)^ According to the descriptive design of the study, no sample calculation and no comparisons were made.

Categorical variables are presented herein according to occurrence and percentages; variables with a normal distribution are presented as the mean and standard deviation. Variables with nonnormal distributions are presented as medians and interquartile intervals. Clinical outcomes are depicted in a stacked bar chart.

## RESULTS

**Figure 1:**
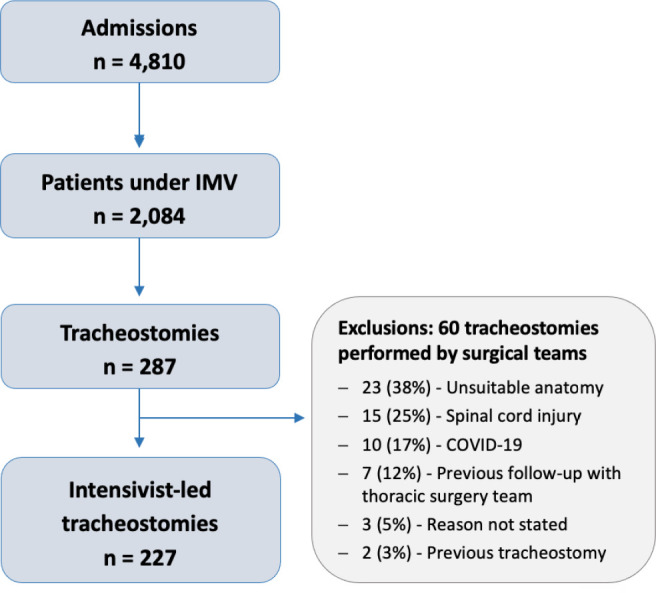
Flow of patients in the study.

From September 2017 to December 2021, there were 4,810 admissions in the study ICUs: 2,084 patients were submitted to MV, and the decision to perform a tracheostomy was made in 287 patients. Of these, 60 were referred to surgical teams (Figure 1). The main reasons to refer a tracheostomy to a surgical team were an unsuitable anatomy (i.e., neck tumor or impossibility of proper positioning), spinal cord injury (confirmed or suspected) with inability to perform neck hyperextension, and COVID-19 infection, due to institutional recommendations.

IMV - invasive mechanical ventilation.

### Sample characteristics

**Figure 2:**
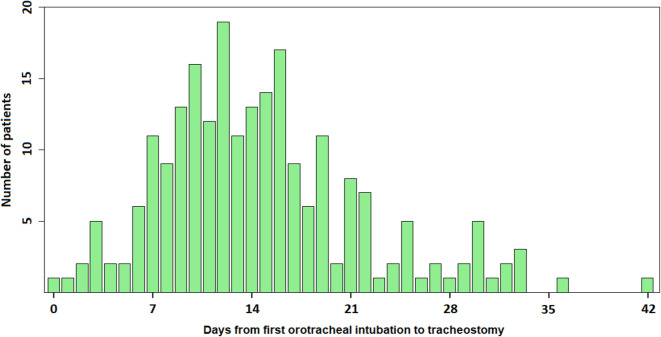
Histogram of days from first orotracheal intubation to tracheostomy.

Table 1 describes the characteristics of the 227 patients undergoing a percutaneous tracheostomy. The mean age was 48 (16) years, and 69% (156/227) were men. The main reason for ICU admission was trauma in 43% (97/227), mostly with traumatic brain injury (84/227; 37%). The main reason to perform a tracheostomy was neurological impairment or inability to protect the airways in 75% (170/227) of the patients. The median time from the first orotracheal intubation to performance of tracheostomy was 14 days (Figure 2). At least one extubation attempt was performed in 33% (75/227) of the patients.

**Table 1:** General characteristics of the patients

Baseline characteristics	
Age (years)	48.06 (16.3)
Male gender, n (%)	156 (68.7)
Weight (kg)	71 [62.9 - 83.8]
Height (cm)	168 [161.1 - 173.5]
Body mass index (kg/m^2^)	25.3 [22.6 - 28.3]
SAPS 3 at ICU admission	59 [50.5 - 67]
Charlson Comorbidity Index	1 [0 - 2]
Diagnosis on admission	
Trauma	97 (42.7)
Traumatic brain injury	84 (37.0)
Facial trauma	45 (19.8)
Cerebrovascular disorder	43 (18.9)
Subarachnoid hemorrhage	17 (7.4)
Cardiac arrest	8 (3.5)
Neuromuscular disorder	15 (6.6)
Acute respiratory failure	33 (14.5)
COVID-19	14 (6.2)
Other	14 (6.2)
Reason for tracheostomy	
Inability to protect the airway	170 (74.9)
Difficult weaning	34 (15.0)
Neuromuscular disorder	14 (6.2)
Airway obstruction	9 (4.0)
Failed attempts at extubation	
0	152 (67.0)
1	50 (22.0)
2	21 (9.2)
> 2	4 (1.8)

SAPS 3 - Simplified Acute Physiology Score 3; ICU - intensive care unit. Results expressed as mean (standard deviation), median [25th to 75th percentiles], or n (%).

Three patients had tracheostomy performed more than 6 weeks from the first intubation (50, 51 and 78 days) and are not shown here.

### Procedural characteristics

Most procedures (173/227; 76%) were performed by intensive care fellows. Airway control was performed by orotracheal tube repositioning with ultrasound guidance in 56.7% of the procedures. The skin incision was transversal in 75.5% (154/227) of procedures. The general characteristics of the procedures are described in table 2.

**Table 2:** Procedural characteristics

Primary operator	
Postgraduate year 3/4 (intensive care fellow)	173 (76.2)
Postgraduate year 1/2 (surgical/clinical resident)	36 (15.9)
Intensivist	18 (7.9)
Airway control method	n = 208
Ultrasound guidance	118 (56.7)
Laryngoscopy (direct or video)	84 (40.4)
Laryngeal mask	3 (1.4)
Ultrasound guidance + laryngoscopy^*^	3 (1.4)
Tracheal dilation technique	n = 217
Griggs forceps	160 (73.7)
Single dilator	57 (26.3)
Skin incision	n = 204
Longitudinal	50 (24.5)
Transversal	154 (75.5)

* The combination of ultrasound and laryngoscopy was not prespecified in the data collection tool and is underreported in this study. Results expressed as n (%).

### Tracheostomy-related adverse events

Most procedures (160/227; 70%) had no complications. Among the complications, the most common was hemodynamic instability, which occurred in almost 10% (22) of the procedures (Table 3). There was no registry of bleeding requiring intervention. In a single procedure, the percutaneous technique was not accomplished, and there was a conversion to the surgical technique (performed by the consultant intensivist). In 7% (16) of the procedures, accidental extubation occurred. In all, except one, repositioning was immediately performed with no consequences. One accidental extubation complicated by hypoxia and a cycle of cardiopulmonary resuscitation after cardiorespiratory arrest reverted without sequelae for the patient.

**Table 3:** Procedure-related adverse events

Adverse events during the procedure	
Hemodynamic instability	22 (9.7)
Extubation during the procedure	16 (7.0)
Cuff puncture	7 (3.1)
Desaturation	6 (2.6)
Incomplete procedure	3 (1.3)
Surgical technique conversion	1 (0.4)
Bleeding	0
Infections	n = 227
Stoma infection	12 (5.3)
VAP in 48 hours	4 (1.8)
Mediastinitis	0
Airway lesions	n = 227
Posterior tracheal wall puncture	4 (1.8)
Pneumothorax or pneumomediastinum	3 (1.3)
False passage	3 (1.3)
Tracheoesophageal fistula	2 (0.9)
Tracheal stenosis	1 (0.4)
Tracheo-innominate artery fistula	0
Other complications	n = 227
Atelectasis	7 (3.1)
Premature decannulation	3 (1.3)
Procedures with any complications	67 (29.5)

VAP - ventilator-associated pneumonia.

There were three postprocedure major complications (2 tracheoesophageal fistulas and 1 case of tracheal stenosis). The two patients with a tracheoesophageal fistula died, and the fistula was considered the direct reason for death.

### Clinical outcomes

**Figure 3:**
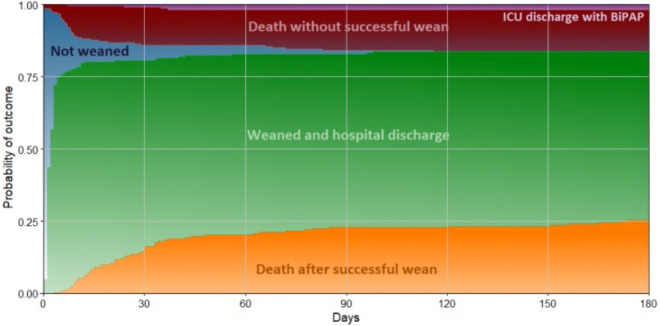
Time course to outcome after tracheostomy.

After the procedure, 80.6% (183/227) of the patients were weaned from MV in a median of 2 [1 - 3] days (Figure 3), and 17.6% (40/227) of the patients died without ever been weaned in a median of 8 [5 - 14] days. Only 1.8% (4/227) of the patients were discharged from the hospital using bilevel positive airway pressure (BiPAP) - all of them had the procedure performed because of a neuromuscular disorder.

Time course of the outcome in 227 patients after percutaneous tracheostomy performed by intensivists. The stacked area chart shows the cumulative percentage of patients who had the outcome (time to hospital discharge not shown).

ICU - intensive care unit; BiPAP - bilevel positive airway pressure.

The ICU mortality was 29.1% (66), while the hospital mortality was 43.6% (99) (Table 4). Only 55/227 (24.2%) of the patients were decannulated before hospital discharge, comprising 43% (55/128) of hospital survivors.

**Table 4:** Hospital outcomes

Length of stay, days	
ICU	26 [19-39]
Hospital	48 [31-72]
Destiny at hospital discharge, n (%)	
Death	99 (43.6)
Discharge home	81 (35.7)
Discharge to a long-term care facility	35 (15.4)
Transfer to another hospital	12 (5.3)

ICU - intensive care unit. Results expressed as median [25th to 75th percentiles] or n (%).

## DISCUSSION

### Main findings

This manuscript describes the experience with tracheostomy performed by intensive care physicians in three ICUs at a quaternary hospital in Brazil after the TRACHUS trial. Almost 80% of the tracheostomies were performed by the intensivist, while most of those performed by a surgical team had a contraindication for performing the bedside percutaneous technique. The main reason to perform tracheostomy was the inability to protect airways in 75% of the patients. We observed a sizable proportion of adverse events during the procedure (approximately 30%), but most of these were inherent to the procedure (such as sedation-related hypotension) and not to the guidance technique. There were three severe procedure-related adverse events.

### Relationship with the literature

The number of complications in our study (29.5%) was comparable with that described in the literature, even including procedures not performed exclusively by the intensive care team. In the TRACHUS Trial,^(13)^ the procedures were performed by intensivists using ultrasound or bronchoscopy; there were 2 major and 32 minor complications among 118 patients (27.1% of the procedures had complications). Lim et al.^(20)^ retrospectively evaluated the outcomes of 458 tracheostomies performed percutaneously by pulmonary intensivists and found that 14.2% of cases had immediate postoperative complications, 7.6% of cases had complications developing within 7 days of the procedure, and 0.4% of cases had long-term complications. In contrast, Romero et al., in their description of bronchoscopy-guided procedures, reported fewer minor complications and no major complications in their cohort.^(21)^

The most common complication in our study was hemodynamic instability, which may be partially attributed to sedative use; in most cases, only transient initiation or an increase in the vasopressor dose was needed. We advise evaluation of hemodynamic optimization (with fluids or vasopressors) before the procedure. Accidental extubation during the procedure was the second most common adverse event (7% of the procedures), which could cause serious adverse events (one patient had hypoxemia and cardiorespiratory arrest). This may be attributed to the absence of bronchoscopy as a method of airway control. This highlights the importance of adequate airway control during tube repositioning away from the puncture line and the need for routine preparation for advanced airway management in cases of tracheal intubation loss. Over time, we began to avoid repositioning the endotracheal tube through ultrasound guidance and favored direct visualization through laryngoscopy. A possible alternative to avoid this complication is to enhance ultrasound imaging by filling the endotracheal tube cuff with saline, as reported by Anand Shankar et al.^(22)^ The number of airway injuries was low (5.7%), and most of them were minor. The incidence of tracheoesophageal fistula was low (0.9%) and comparable to the literature,^(13)^ although there are reports from the literature of such fistulas not occurring with the use of bronchoscopic guidance.^(21)^

The median time to tracheostomy in our cohort was 14 days. The ideal time to perform a tracheostomy is still not clear in the literature. Terragni et al.^(23)^ randomized 600 patients to be submitted to an early tracheostomy (within 6 - 8 days of MV) or to a late tracheostomy (after 13 - 15 days) and found no difference in the incidence of VAP or mortality. In the TracMan Trial, Young et al.^(7)^ randomized 455 patients to be submitted to a tracheostomy performed early (within 4 days) or late (after 10 days) and found no difference in mortality or antibiotic use. In the SETPOINT 2 Trial, Böse et al.^(24)^ randomized 382 patients with severe acute ischemic or hemorrhagic stroke to be submitted to an early tracheostomy (before 5 days of intubation) or to a late tracheostomy (after 10 days) and found no difference in functional outcome at 6 months.

In this study, no bleeding that demanded intervention (surgical control or transfusion) was registered, which may be explained by ultrasound guidance allowing the identification of vessels anterior to the trachea and changes in the puncture site. In a randomized trial with 80 patients submitted to percutaneous tracheostomy, 40 of whom received ultrasound guidance, Sarıtas et al. found only one (2,5%) hemorrhage complication, which was considered minor.^(25)^

Despite these complications, the clinical outcomes of our cohort were better than those previously reported in Brazilian studies,^(26)^ with a 29% ICU mortality and a 43% hospital mortality, but higher than an American cohort that reported an 18.4% mortality.^(27)^ The ICU length of stay (26 days) was also similar to the findings reported by Nishi et al., namely, 28 days.^(27)^

### Strengths and limitations

Our study has some strengths, one of which is the prospective collection of data using a tool a priori designed for this use, which minimized collection bias and allowed a reliable analysis of complications. Data missingness was low and likely did not result in measurement error.

One possible limitation of this study is that the technique for tracheal dilatation was performed according to the kit that was available in the hospital at the time of the procedure, along with the 4-year time span and the fact that the tracheostomies were performed by different providers. However, given that this can be considered a follow-up phase IV study, this enhances the effectiveness assessment and potential risks of the procedure when performed in a real-world scenario outside of a clinical trial. Additionally, there was no formal method of screening airway lesions, other than a chest X-way. This may have led to an underreporting of airway lesions. On the other hand, a diagnostic work-up was performed if the patient had any symptoms that suggested the presence of an airway lesion. Therefore, if there was any underreporting of airway lesions, those lesions were probably minor and caused no repercussions to the patients. The definition of hemodynamic instability (the need to start or increase the dose of vasopressors) was subjective, and some episodes of hypotension may have been assumed to be transient and left untreated; however, the clinical meaning of sedation-related transient hypotension episodes is unclear and unlikely to affect clinical outcomes. Although we prospectively collected the data to allow a more precise estimation of adverse events, some data collection was not prespecified (such as using laryngoscopy combined with ultrasound guidance as a method of airway control), and other data necessitated medical record revision to avoid missing data. Finally, these results are from a single center, so caution should be taken when generalizing the results to other contexts. Nevertheless, these findings represent the results from different ICUs and different intensivists performing the procedure in the context of intensivist training, representing a desired variability of usual practice.

### Implications for practice, education and policy

The number of complications, which was comparable to that in the literature, suggests that the intensivist-led procedure is likely safe, although with caveats that need to be considered. Intensivists can be taught and can learn to safely perform ultrasound-guided procedures in patients without contraindications to the percutaneous technique.

Even though the risk of serious adverse events is low, they do occur, which highlights the fact that tracheostomy is not an innocuous procedure and that the decision to perform it must be precise. Furthermore, in complicated procedures, the threshold to investigate other complications should be low.

We also believe our experience, which comprises many intensivists trained during fellowship to perform tracheostomy, can be replicated at other institutions to allow intensivist-led tracheostomy to be performed where necessary. Although we gained experience through time to perform ultrasound-guided tracheostomy, we also believe that the availability of a bronchoscope in the ICU would be beneficial for intensivists to conduct safer tracheostomy procedures and other airway management techniques. This is a gap in the Brazilian critical care community that requires policy changes to allow its more widespread incorporation and training.

## CONCLUSION

Intensivist-led ultrasound-guided percutaneous tracheostomy is feasible outside of a clinical trial context, with outcomes and complications comparable to those in the literature. Intensivists can acquire this competence during their training but should be aware of potential complications to enhance procedural safety.
